# Pharmacist-driven antimicrobial stewardship interventions in patients with COVID-19: a scoping review

**DOI:** 10.1007/s11096-023-01574-0

**Published:** 2023-05-10

**Authors:** Z. G. Nasr, W. Elamin, M. Basil, K. Eljaaly

**Affiliations:** 1 College of Pharmacy, QU Health, Qatar University, Doha, Qatar; 2 Faculty of Pharmacy, King Abdulaziz University, Jeddah, Saudi Arabia

**Keywords:** Antimicrobial Stewardship, COVID-19, Pharmacist

## Abstract

**Background:**

Coronavirus Disease 2019 (COVID-19) is a highly infectious disease that can be treated with antivirals in addition to other antimicrobials in cases of secondary or concomitant infections. This creates potential for antimicrobials misuse, which increases antimicrobial resistance (AMR). Pharmacists are known to undertake prominent roles in combatting AMR.

**Aim:**

The aim of this review was to characterize pharmacist-driven interventions that have been performed in patients with COVID-19 globally and describe their impact on antimicrobial use.

**Method:**

We followed the Joanna Briggs Institutes manual framework for scoping reviews in our study. Studies that reported antimicrobial stewardship (AMS) interventions performed by pharmacists in COVID-19 patients were included. Articles that did not report outcomes or did not mention pharmacists in the intervention were excluded. Restrictions included English-only articles from inception date until June 2022. Articles were searched from four databases.

**Results:**

Eleven publications were included in the review. The most common AMS intervention was pharmacist-driven interventions reported in 63.2% of all studies, followed by guideline development and application (26.3%), and medication supply coordination (10.5%), respectively. The outcomes of the interventions were difficult to compare but showed a reduction in antimicrobial use and prevention of adverse drug reactions with a relatively high acceptance rate from physicians.

**Conclusion:**

Pharmacists played an important role in performing AMS-related interventions in COVID-19 patients and helped in the fight against the worsening of AMR during the pandemic. The impact of pharmacist-driven AMS interventions in patients with COVID-19 seemed to be positive and improved outcomes related to antimicrobial use.

**Supplementary Information:**

The online version contains supplementary material available at 10.1007/s11096-023-01574-0.

## Impact statements

- Pharmacist-driven AMS interventions can improve outcomes related to antimicrobial use in patients with COVID-19.
- Pharmacists are well positioned health care professionals who play a vital role in the fight against AMR during the COVID-19 pandemic.

## Introduction

The World Health Organization (WHO) declared the Coronavirus Disease 2019 (COVID-19) outbreak a global pandemic in March 2020 [1]. Due to the viral nature of COVID-19, the United States Food and Drug Administration (FDA) has currently approved the use of the broad-spectrum antiviral remdesivir in addition to giving Emergency Use Authorizations (EUA) for molnupiravir and ritonavir-boosted nirmatrelvir [2]. Given the pathogenesis of the disease, patients infected with the virus can develop secondary bacterial or fungal infections that necessitate the employment of antimicrobials for treatment [3]. A recent meta-analysis showed that only 7% of hospitalized patients with COVID-19 were reported as having an evident bacterial co-infection with more than 90% of patients receiving antimicrobials [4]. In addition, early administration of antibiotics did not impact mortality in critically ill patients with COVID-19 [5], and the use of broad-spectrum antibiotics like carbapenems early during treatment did not impact treating the superinfection and might increase the emergence of resistant microbiological strains [6]. This creates the potential of misusing antimicrobial agents for unknown indications which would subsequently contribute to an increase in antimicrobial resistance (AMR) [7]. AMR continues to be a public health threat and it is predicted to cause over 10 million deaths by 2050 if no appropriate action plans were put in place [8]. In an effort to reduce AMR, antimicrobial stewardship (AMS) is means to better measure and improve the prescribing and use of antimicrobials as defined by the Centers for Disease Control and Prevention (CDC) [9]. In order to guide healthcare institutions in the implementation of antimicrobial stewardship programs (ASPs), the CDC released an updated list of core elements in 2019 focusing on “Pharmacy Expertise” [9]. In fact, pharmacists, especially infectious diseases (ID) pharmacists, play vital roles in AMS [9]. They lead, or co-lead ASPs, educate healthcare professionals on AMS, develop local and institutional guidelines and protocols, monitor and measure the use of antimicrobials, assess antimicrobial regimens for drug-related problems, perform pharmacokinetic drug monitoring, adopt alternative dosing strategies and perform allergy assessment, counsel on the use of antimicrobials, review full antimicrobial regimen (right drug for the right indication, dose, route, frequency, and duration of therapy), adjust doses for organ dysfunction, streamline agents based on culture and sensitivity report, switch from intravenous (IV) to per oral (PO) formulations when appropriate, amongst other interventions [10, 11]. Given the aforementioned roles, pharmacists’ involvement in overseeing antimicrobial therapy in COVID-19 patients becomes critical. Regrettably, there have already been reports in literature emphasizing the need for enhanced AMS efforts. For instance, a meta-analysis reported an increased prevalence of Multi-Drug Resistant Organisms (MDROs) in COVID-19 wards [12], as well as an increased prevalence of QTc prolongation in COVID-19 patients secondary to unjustified use of hydroxychloroquine/chloroquine therapy or in cases of drug-drug interactions [13, 14]. For the past 2 years, much literature has documented the general roles pharmacists are playing in the ongoing COVID-19 pandemic [15, 16]. Nevertheless, AMS- related interventions by pharmacists and observed outcomes have not been the focus of those articles. There have also been publications about AMS interventions in COVID-19 patients [17]. However, such articles have either presented a general perspective on the matter or showcased hospital- or country-specific interventions [17].

### Aim

The aim of this scoping review was to characterize pharmacist-driven interventions that have been performed in patients with COVID-19 globally and describe their impact on antimicrobial use.

## Method

We followed the Joanna Briggs Institutes manual framework for scoping reviews when conducting our study [18]. A protocol was developed *a priori* and was followed without deviation.

### Study selection

#### Participants

Studies conducted on patients with COVID-19 receiving antimicrobials were included in this review.

#### Concept

We adopted the concept of AMS interventions performed by pharmacists. The classification was adopted from the CDC 2019 core elements of antibiotic stewardship or labeled by the investigators as deemed appropriate [9].

#### Context

Studies conducted in a clinical setting with no restriction to region, country or geographic area were considered for this review.

#### Types of sources

Studies with any type of research design were considered.

### Search strategy

A search of PubMed, SCOPUS, EMBASE, and Google Scholar until June 2022 was conducted. MeSH terms, keywords, and text words were combined using Boolean operators. Search terms included terms that were related to COVID-19, antimicrobial agents, stewardship, pharmacist, and roles. The search strategy can be found in Table 1 of the supplementary material. All search terms were limited to Title/Abstract. The electronic search was supplemented with a manual search of the reference lists from identified relevant studies.

**Table 1:** Characteristics of included studies

Author/Country/Year	Pharmacist-driven	Medication supply coordination	Developing guidelines and implementation
Al-Quteimat et al. UAE, 2022 [20]	✓		
Collins et al. USA, 2020 [21]	✓	✓	✓
Gourieux et al. France, 2021 [22]	✓		✓
Mazzone et al. USA 2021 [23]	✓	✓	
Murgadella-Sancho et al. Spain, 2022 [24]	✓		✓
Ng et al. Singapore, 2021 [25]			
Perez et al. France, 2022 [26]	✓		✓
Pettit et al. USA, 2021 [27]	✓		✓
Peterson et al. USA, 2022 [28]	✓		
Schmid et al. Germany, 2022 [29]	✓		
Wang et al. China, 2021 [30]	✓		

### Extraction of results and data synthesis

The search results were then transferred to Rayyan online software [19] to facilitate the screening process. After removing duplicates, articles were screened for eligibility independently by two investigators (WE and MB) and were checked for accuracy by a third investigator (ZN). Articles were included if they reported at least one clearly described antimicrobial stewardship intervention performed by pharmacists or AMS team including a pharmacist. To be included, the intervention should have been performed in COVID-19 patients or related to antimicrobial agents used for COVID-19. Studies that did not include pharmacists in the interventions and those that did not report any outcomes were excluded. Additionally, we excluded any article that was not available as full text or in English language or published before January 2020. Discrepancies between investigators were resolved by discussion. Data were extracted by WE and MB using an extraction spreadsheet and were reviewed by ZN for verification. The data extraction tool can be found in Table 2 of supplementary material. All investigators met via videoconference on multiple occasions over 2 months to discuss articles and interpret findings. The included interventions were then objectively analyzed, compared, and classified. Furthermore, reported outcomes were also depicted.

**Table 2:** Impact of AMS interventions and reported outcomes

Study	Impact/outcome
Al-Quteimat et al. [20]	Dose optimization of antimicrobials was the most common AMS-related intervention reported 94.7% of interventions were accepted by physicians.
Collins et al. [21]	Adjustment of the use of antimicrobials for the treatment of COVID-19 was the most common outcome of AMS-related pharmacist intervention (15.2%).
Gourieux et al. [22]	Adjustment of wrong treatment duration (54.2%), followed by torsadogenic drug interactions (23.7%), and incorrect antimicrobial dosing (10.2%) were the most common reported outcomes. The acceptance rate of physicians was 81.4%.
Mazzone et al. [23]	Interventions led to the prevention of the unnecessary use of restricted antimicrobials in 33.7% of the patients 304 patients received remdesivir with the coordination in compounding from the local hospital.
Murgadella-Sancho et al. [24]	Reduction in antimicrobial consumption in 2020 measured as DDD; (57.8 DDD/100 bed days) compared to 2019 (64.7 DDD/100 bed days) (*p* = 0.045).
Ng et al. [25]	Despite the decrease in the number of pharmacists who are responsible for prospective audits, the number of recommendations and the acceptance rate were maintained as they were prior to the pandemic.
Perez et al. [26]	Physicians' acceptance rate for pharmacists’ interventions was 88.5% with a great reduction in ADRs because of pharmacists’ interventions.
Pettit et al. [27]	Prescribing rate of azithromycin, cefdinir, and ceftriaxone was significantly decreased in the post-intervention group (post guideline development) when compared to that of the pre-intervention group (*p* < 0.001, *p* = 0.001, *p* = 0.005, respectively). Initiation of antibiotics based on guideline recommendations was significantly higher in the post-intervention group compared to the pre-intervention group (*p* = 0.001).
Peterson et al. [28]	The average monthly percent changes of antibiotics in hospitals utilizing different ASPs showed trends that were similar to before the pandemic.
Schmid et al. [29]	In 2021, the consumption declined to 147.8 recommended daily doses (RDD)/100 patient days (PD) in comparison to the year before (155.4 RDD/100 PD). Moreover, the expenditure also decreased to 75,292 EUR in 2021 compared to the year before (76,764 EUR).
Wang et al. [30]	The acceptance rate of physicians for pharmacists’ interventions was 95.5%.

## Results

### Study selection

**Fig. 1:**
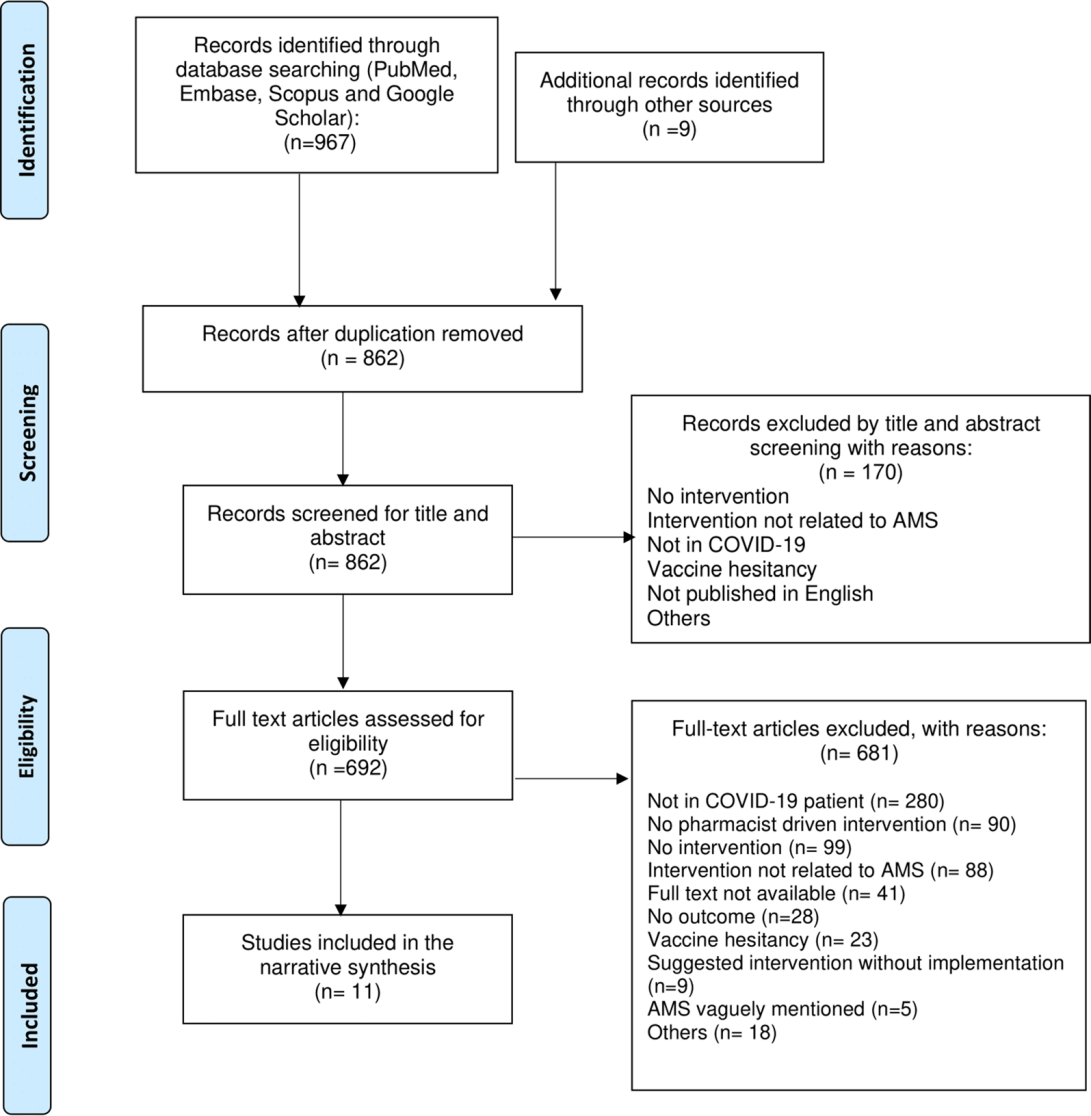
Flowchart of included studies

As shown in the PRISMA extension for scoping review (PRISMA-ScR) flow diagram (Fig. 1), electronic database searching resulted in 967 hits. After a full-text review, we identified 11 studies that were included in the review.

### Characteristics of included studies and types of AMS interventions

**Fig. 2:**
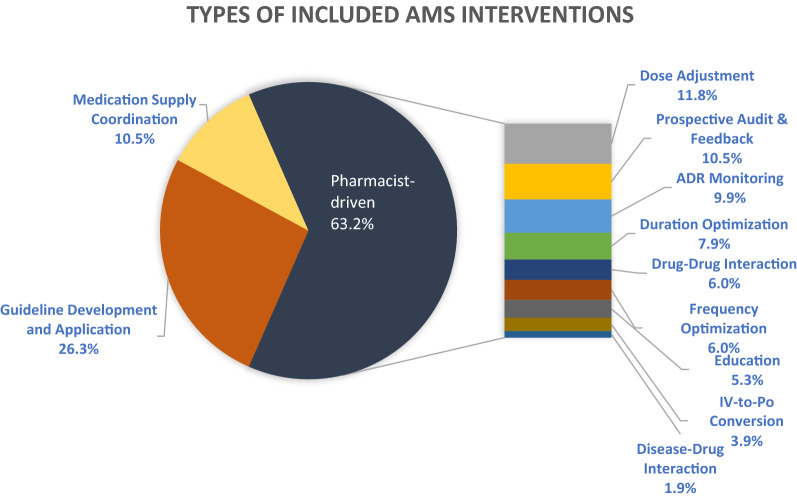
Percentages of studies reporting each of the intervention types

Table 1 illustrates the types of interventions in each study. Figure 2 showcases a detailed observation of the interventions included in our review performed by pharmacists. The most common AMS intervention was pharmacist-driven interventions reported in 63.2% of all included studies, followed by guideline development and application (26.3%), and medication supply coordination (10.5%), respectively.

#### Pharmacist-driven interventions

Ten of the 11 included studies reported implementing at least one of the pharmacist-driven interventions [20–24, 26–30]. The identified pharmacist-driven interventions were proper selection of antibiotics, switching from IV to PO administration, dose/frequency adjustment, duration optimization, Adverse drug reactions (ADRs) monitoring, and identifying drug-drug interactions.

In four studies, pharmacists aided in drug selection and recommended the appropriate antimicrobial based on culture results and advised switching from IV to PO where applicable [23, 26, 28, 29]. Other studies reported that pharmacists adjusted antimicrobial dosing to fit therapeutic ranges in order to avoid ADRs because of high doses or impaired renal function [23, 30]. For instance, dose adjustment of lopinavir/ritonavir, hydroxychloroquine, azithromycin, penicillin, and macrolides was performed [22, 26]. Moreover, therapeutic drug monitoring was conducted for certain antimicrobials such as vancomycin and voriconazole due to sub/supratherapeutic drug concentrations [30]. Five studies described pharmacists’ interventions related to ADR monitoring [21–23, 29, 30]. Such interventions included daily monitoring of hepatic function with remdesivir and QT prolongation with lopinavir/ritonavir and azithromycin [21–23].

In terms of optimizing the treatment duration, pharmacists in one study set the duration of treatment in the electronic system to be aligned with a COVID-19 developed protocol [24]. Similarly, in another study, pharmacists kept track of the days of therapy (DOT) of antibacterial and antiviral drugs to minimize unnecessarily prolonged use [21]. Furthermore, pharmacists adjusted the doses and/or frequency of dosing of antimicrobial regimens including lopinavir/ritonavir, hydroxychloroquine, and azithromycin based on guidelines [22].

Prospective audit and feedback was performed by pharmacists on a daily basis in COVID-19 patients in two studies [22, 25]. The first study coupled the audit and feedback with continuous antimicrobial surveillance [22]. The other study relied on the Global Point Prevalence Survey (PPS) to compare the prevalence of antimicrobial prescribing before COVID-19 versus during COVID-19 with a focus on broad-spectrum antibiotics such as piperacillin/tazobactam and meropenem, and assessed whether shortages in AMS team’s manpower affected the number of recommendations and acceptance rate [25]. Lastly, pharmacists educated medical teams during virtual rounds daily, in addition to educating the other healthcare providers and emergency department staff on the appropriate use and selection of antimicrobials [24, 27]. COVID-19 patients also received education on their treatment plan [24].

#### Guideline development and application

Four studies included interventions related to guideline development [21, 22, 24, 27] and one study related to guideline implementation [26], which aided in establishing clear diagnostic criteria, treatment algorithms and drug safety monitoring. ID team, ID or AMS pharmacists, residents, and students developed hospital-specific guidelines for the management of patients with COVID-19 in an effort to maximize the evidence review process in compliance with the institution’s AMS policy [21, 22, 24, 27]. Additionally, pharmacists followed up, documented, and ensured that drug prescribing followed the developed guideline [26].

#### Medication supply coordination

Interventions related to medication supply coordination were discussed in two studies [21, 23]. A field hospital report indicated minimizing waste and preventing potential remdesivir shortage was performed through a working strategy by a compounding pharmacist [23]. In another study, pharmacists proactively developed strategies to minimize resource limitations by ordering sufficient stocks of antimicrobials to meet predicted pandemic-wave needs [21]. Moreover, to prevent shortages in personal protective equipment and staff exposure to the virus; automated dispensing cabinets’ stocks were increased and the workflow in the IV room was adjusted [21].

### Impact of AMS interventions

Pharmacist-driven AMS interventions in COVID-19 patients led to relatively positive outcomes and showed good impact on antimicrobial use. This was evident by adjustment of poor antimicrobial use [21, 22], prevention of unnecessary antimicrobial use and decreased prescribing rates [23, 27], reduction in antimicrobial consumption [24, 28, 29], high physician acceptance rates [20, 22, 25, 26, 30], adjustment of drug-drug interactions [22], and better adherence to guidelines [27]. For better visualization, a summary of the impact or outcomes of AMS interventions can be found in Table 2.

## Discussion

The purpose of this review was to explore AMS interventions and actions of pharmacists in COVID-19 patients and to describe their outcomes. Although most AMS interventions were pharmacist-driven interventions, other activities included guideline development and application, and medication supply coordination.

Several limitations should be noted in our review. There seems to be a deficit in reporting pharmacist-driven interventions in COVID-19 patients. Even when reported, the description of the intervention was ambiguous and lacked the needed details to properly understand or replicate. Another shortfall is the scarcity and inconsistency of the reported outcomes. This might have led to a low number of articles included. In addition, the burdens that were associated with the pandemic could have also played a part in the low number of included studies. In fact, the consequences of COVID-19 unfortunately overworked healthcare professionals and overloaded medical institutions [31]. It also led to the diversion of resources away from ASPs towards COVID-19 care which could potentially reduce the activities of ASPs [32]. Furthermore, it is also possible that due to the consequent reduction in resources such as time and funds, pharmacist-driven interventions and outcomes might have been performed and reported in some institutions, but were not published. During the COVID-19 pandemic, the utilization of antimicrobials could have been impacted by drug shortages and/or limitation of resources. Therefore, the findings of published studies should be interpreted with caution given the negative impact of those limitations on internal validity. While not ideal, the search was limited to articles in English due to resource limitations. Lastly, several studies focused on pharmacists’ general interventions and their role in the COVID-19 pandemic rather than specific AMS-related pharmacist interventions. However, this review still presents a comprehensive narration of articles discussing pharmacist-driven interventions and outcomes reported in COVID-19 patients.

Most AMS interventions performed by pharmacists followed recommendations provided by the CDC and WHO [9, 33]. Moreover, the reported interventions also fall in line with Garau et al.’s study about the role of pharmacists in ASPs [34]. In the study, pharmacists performed prospective audits and feedback, ensured surveillance, performed IV to PO antimicrobial switching, optimized antimicrobial duration of therapy, and educated healthcare professionals and patients on antimicrobials used in COVID-19 [34].

Pharmacist-driven interventions represent a critical part of ASPs to ensure the safe and effective use of antimicrobials [10]. It was noted that remdesivir was the source for most of the pharmacist-driven interventions for COVID-19 specific treatment. It is still recommended to be used in patients with COVID-19 [2]. However, it requires frequent hepatic monitoring as it carries a risk of hepatotoxicity [2]. Despite not being recommended, several AMS interventions related to HCQ use were notable in the review. Serious ADRs including prolongation of the corrected QTc interval could lead to potentially fatal arrhythmia of Torsades de Pointe requiring frequent monitoring by pharmacists [35]. Other important pharmacist-driven intervention included IV to PO therapy conversion which is associated with the reduction of hospital stays, hospital-acquired infections, burden on hospital staff, and infections related to IV therapy [36]. Also, it is important for healthcare professionals to follow up on polymerase chain reaction testing results and stop antimicrobials if results came back negative [37]. Similarly, empiric antimicrobials should be re-assessed and de-escalated based on culture and sensitivity reports, or based on other clinical re-assessments and diagnostic criteria [36].

Interventions related to clinical guideline/protocol development help in antimicrobial use optimization [9]. Facility-specific guidelines should be continuously updated, disseminated, educated about, and modified based on new emerging data [9]. Prospective audit and feedback necessitates an assessment of appropriate antimicrobial use during the pandemic. It also requires proper workflow and efficient communication among several departments within the institution including IT and microbiology. This can help improve documentation in electronic medical records [9]. Activities related to drug supply maintenance and mitigation of drug shortages are essential components of AMS [33]. Medication supply coordination has been deemed important by many organizations including the WHO [33]. Lack of access to antimicrobials in some parts of the globe can lead to more deaths than AMR itself. Thus, it is important to address medications at risk of shortage [38]. In their study, Collins et al. reported that pharmacists had the responsibility to deal with and respond to shortages in medications and medical supplies [21]. Moreover, the CDC considers education an important element to part of the improve antimicrobial use within institutions, whether it was delivered through virtual presentations, educational materials, or via electronic communications [9]. In addition, information used for education should be updated regularly based on new emerging data [39].

Despite being too different to properly compare, several outcomes related to AMS interventions seemed to have a positive impact on antimicrobial use as they led to better decision-making, and reduction in DOT, defined daily dose (DDD), and ADRs. A systematic review with a narrative synthesis by Monmaturapoj et al. evaluated pharmacist-driven interventions in inpatient settings [40]. Interventions included education, prospective audit and feedback, reminders, and restriction [40]. Their findings revealed that pharmacist-driven interventions were associated with a reduction in antimicrobial use [40]. This is consistent with findings of Pettit et al.’s study which showed reduced antimicrobial prescribing while developing guidelines, implementing education and pharmacist-driven interventions [27].

There have already been reports in the literature emphasizing the need for enhanced AMS efforts [43]. A survey conducted in the UK showed that more than 60% of the participating centers claimed that COVID-19 negatively affected routine AMS activities by decreasing the number of the multidisciplinary team meetings and medical rounds [41]. It is recommended to consider AMS a priority measure in disaster response to pandemics. More specifically, health institutions should employ AMS interventions in patients with COVID-19 as part of their ASPs. The involvement of pharmacists in those programs should increase as they are well positioned to perform valuable interventions [42]. Improvement in the reporting of both interventions and outcomes is vital as it would aid in characterizing the impact of pharmacist-driven AMS interventions in patients with COVID-19, as well as facilitating their reproducibility in other institutions or countries. This is not only important during the ongoing COVID-19 pandemic but could potentially be beneficial in planning efficient AMS mitigation measures in case a similar outbreak ever emerged.

## Conclusion

ASPs can support pandemic response efforts. Pharmacists played an important role in performing AMS-related interventions in COVID-19 patients and helped in the fight against the worsening of AMR during the ongoing pandemic. There seem to be several ASP opportunities that could be performed during the pandemic by pharmacists. Although difficult to aggregate, the impact of pharmacist-driven AMS interventions in patients with COVID-19 appears to be positive with improved outcomes related to antimicrobial use.

## Supplementary Information

Below is the link to the electronic supplementary material.

Supplementary file1 (DOCX 28 kb)
