# Supplementary material for: Pharmacist-driven antimicrobial stewardship interventions in patients with COVID-19: a scoping review
Source: Int J Clin Pharm. 2023 May 10;45(3):613–21. doi: 10.1007/s11096-023-01574-0 (PMC10171144; doi:10.1007/s11096-023-01574-0)
Supplement: Supplementary file 1 — Supplementary file1 (DOCX 28 kb) [file 11096_2023_1574_MOESM1_ESM.docx]

**Search strategy**

The full search that was conducted for each database is described below:

1. **PubMed:**

COVID-19"[Mesh] AND ('antiinfective agent' OR antiviral OR antifungal OR antiprotozoal OR antimicrobial OR antibacterial OR antibiotic) AND (stewardship program OR stewardship OR education OR 'infection control') AND (pharmacist led OR pharmacist-led OR pharmacist)

1. **Embase:**

('coronavirus disease 2019') AND (anti-infective agent OR antiviral OR antifungal OR antiprotozoal OR antimicrobial OR antibacterial OR antibiotic) AND (stewardship program OR stewardship OR education OR 'infection control') AND (intervention) AND (pharmacist led OR pharmacist-led OR pharmacist)

1. **Scopus**

(antibiotic* OR 'antiinfective AND agent' OR antiviral OR antifungal OR antiprotozoal OR antimicrobial OR antibiotic) AND (stewardship AND program OR stewardship OR education OR 'infection AND control' ) AND (intervention) AND ( pharmacist AND led OR pharmacist-led OR pharmacist OR pharmacy) AND ( covid 19 OR coronavirus OR pandemic OR severe AND acute AND respiratory AND syndrome OR sars AND cov OR sars-cov-2 ) AND role*

1. **Google Scholar:**

(antibiotic* OR antiinfective agent OR antiviral OR antifungal OR antiprotozoal OR antimicrobial OR antibacterial) AND (stewardship program OR stewardship OR education) AND (pharmacist-led OR pharmacist) AND (COVID-19 OR corona virus OR pandemic OR SARS-CoV-2) AND role*

**The term “role” was not included in the search strategy of two databases (PubMed and Embase) as it resulted in limiting the number of relevant studies.*

Table 1. Example of PubMed’s detailed search strategy

| Concept | Search Terms | Boolean Operators |
| --- | --- | --- |
| 1. COVID-19 | - 1. COVID-19 [MeSH] | AND |
| 1. Antimicrobial agents | - 1. Antibiotic* | OR |
|  | - 1. Anti-infective AND agent | OR |
|  | - 1. Antiviral* | OR |
|  | - 1. Antiprotozoal* | OR |
|  | - 1. Antimicrobial* | OR |
|  | - 1. Antibacterial* | OR |
|  | - 1. Antibiotic* | AND |
| 1. Stewardship | - 1. Stewardship AND program* | OR |
|  | - 1. Stewardship | OR |
|  | - 1. Education | OR |
|  | - 1. Infection AND Control | AND |
| 1. Pharmacist | - 1. Pharmacist AND led | OR |
|  | - 1. Pharmacist-led | OR |
|  | - 1. Pharmacist* | AND |
| 1. Role | - | - |

**Table 2. Data Extraction Tool**

| Article # | Title | Authors/Year | Country | Design | Duration | Population | Type of Intervention | Intervention(s) Summary | Outcomes |
| --- | --- | --- | --- | --- | --- | --- | --- | --- | --- |
| 1 | The Second Wave of COVID-19:  Clinical Pharmacy Services During  a Field Hospital Operation | Mazzone, J, et al/ 2021 | USA | Hospital report | Dec 2020 -March 2021 | COVID-19 patient (not specified if inpatient or outpatient) | Pharmacist-driven (IV-to PO, dose adjustment, ADR monitoring)  Other: Medication supply coordination  AM review | 1. Pharmacists reviewed all the antibiotic therapy  2. They performed renal dose adjustments  3. They converted from IV to oral for applicable medication  4. Once the ID team approved remdesivir, the pharmacist ensured that it is ordered with a 30-ml bolus NS following each administered dose  5. Pharmacists at the field hospital coordinates with inpatient pharmacy services at the local hospital campus to compound remdesivir doses for the number of patients receiving treatment (they need to maintain enough doses to treat every patient for 24 hours while minimizing waste.  6. pharmacists monitored daily the renal function and hepatic enzyme for patient on remdesivir  Pharmacists implemented 668 interventions: AMS was the most performed intervention (34.2%) and was as following  1. Restricted antibiotics (33.7%)  2. Other (0.3%)  3. Empiric therapy recommendation (0.3%). | The interventions led to the prevention of the unnecessary use of restricted antimicrobial in 33.7% of the patients. |
| 2 | Reducing the use of empiric antibiotic  therapy in COVID-19 on hospital admission | Pettit N, et al/ 2021 | USA | Retrospective cohort study | March- April 2020 | Patients admitted to the hospital with COVID-19 who received one or more doses of empiric antibiotics within 48 hours of administration for community acquired bacterial pneumonia (CABP)  - Total of 246 patients were included in the study divided as:  - 76 patients in the pre-intervention group  - 170 patients in the post-intervention group | Guideline Development, Education (staff) | The hospital ID consult team included infectious disease/antimicrobial stewardship pharmacist and ID physician were they:  1. participated in preparing the hospital inpatient COVID-19 management guideline  2. Provided daily education and recommendation about the use of antibiotics for the inpatient COVID-19 medical team in the virtual rounds.  3. Provided education for emergency department staff.  4. Counselled patients on the antibiotics therapy and COVID-19 managements plan. | 1. Certain antibiotics (azithromycin, Ceftriaxone, Cefdinir) showed a statistically significant decreased in the rate of prescribing in the post-intervention group compared to the pre-intervention group.  2. Median antibiotic duration was significantly less in the post-intervention group (1.3 days shorter) compared to the pre-intervention group  3. Duration of atypical antimicrobial coverage decreased significantly in the post-intervention group (2.3 days shorter) compared to the other group  4. No differences were observed between the two groups in term of number of C. difficile infection, the need for antibiotic re-initiation, length of stay, all-cause readmission rate and mortality rate. |
| 3 | Impact of the strategies implemented by an antimicrobial stewardship program on the antibiotic consumption in the coronavirus disease 2019 (COVID-19) pandemic | Murgadella-Sancho, A, et al/ 2022 | Spain | Pre/post study | 2019 (pre-COVID-19) and 2020 (during COVID-19) | Non-critically ill COVID-19 patients where comparison of antibiotic consumption before and during COVID-19 period was made, and investigation of the effectiveness of antimicrobial stewardship interventions applied by AMS team during the COVID-19 period | Guideline Development, Education (staff), Pharmacist-driven intervention (duration optimization) | Pharmacists were members of the AMS team where they:  1. Developed guidelines for the management of COVID-19 patient that provide information regarding the recommended antibiotic and the appropriate duration (ceftriaxone for 5 days and azithromycin for 3 days).  2. Educated other health care provider about the recommend antibiotic duration of these medications  3. Fixed the durations option in the electronic system to the duration specified in the protocol  4. Reviewed antimicrobial prescriptions before verification. | - The monthly consumption of antibiotic during hospitalization was expressed as defined daily dose (DDD) per 100 bed days  - Consumption in 2020 (57.8 DDD/100 bed days) was lower than 2019 (64.7 DDD/100 bed days) (statistically significant, t=0.045), except for March were the antibiotic prescribing slightly increased.  - From March-April 2020 the most used antibiotics were Azithromycin (16.1 DDD/100 bed days) and Ceftriaxone (5.8 DDD/100 bed days).  - In May, piperacillin-tazobactam was the highest antibiotic consumed (5.4 DDD/ 100 bed days) |
| 4 | Analysis of Pharmacist Interventions in Adult COVID-19 Patients Admitted to a Tertiary Care Hospital | Al-Quteimat O, et al/ 2022 | UAE | A retrospective chart review to analyze the documented clinical pharmacists' interventions in patients with confirmed COVID-19. | 4 months | admitted adult patients with confirmed COVID-19 diagnosis | Pharmacist-driven (dose optimization, duration, discontinuation, IV to PO) and de-escalation | - Pharmacists reported 484 interventions with antimicrobial stewardship interventions being the most reported (149, 30.8%). Those included dose optimization, de-escalation, duration of therapy, and discontinuation. - The most frequently stewarded antibiotics were piperacillin–tazobactam, hydroxychloroquine, and ritonavir-lopinavir. | -The physician acceptance rate to the interventions was 94.7%.  -The most commonly reported outcomes of interventions were optimized therapy 60%, avoided adverse event 18%, improved communication 13%, and saving costs 9%. |
| 5 | Effects of coronavirus disease 2019 (COVID-19) pandemic on antimicrobial prevalence and prescribing in a tertiary hospital in Singapore | Ng TM, et al/ 2021 | Singapore | Retrospective study comparing 5 cross-sectional surveys conducted in years 2015, 2017, 2018, 2019 and 2020 | 5 years (before and after COVID-19) | COVID-19 patients on antibiotic therapy | Prospective review and feedback | - Prospective audit and feedback were done by a team that included 4 full-time equivalent pharmacists (previously 5 before COVID-19) who provided daily reviews on piperacillin-tazobactam, carbapenem, and ciprofloxacin. | -There was no increase in antimicrobial prescribing, and no significant differences in antimicrobial prescribing quality indicators.  -The use of Amoxicillin and piperacillin with beta-lactamase inhibitors as well as ciprofloxacin decreased in 2020; while the use of carbapenems increased. |
| 6 | Analysis of clinical pharmacist interventions in the COVID- 19 units of a French university hospital | Perez M, et al/ 2022 | France | A prospective cohort study | 1 month | COVID-19 patients | Pharmacist-driven (IV-to PO, dose-optimization), Following guidelines | - Clinical pharmacists had documented 188 interventions. Interventions related to antimicrobial agents (n=33, 17.6%). 23 of those interventions were patients with confirmed positive COVID-19; 17 for penicillin and 5 for macrolides. - The pharmacy-based interventions included IV to PO conversions, dose adjustment, and drug-drug interactions such as the co-prescription of piperacillin/tazobactam with amoxicillin/clavulanic acid - Pharmacists also ensure the conformity of prescriptions with guidelines | -The interventions led to a significant reduction in drug related problems such as adverse events. |
| 7 | Perspectives from the frontline: A pharmacy department’s response to the COVID-19 pandemic. American Journal of Health-System Pharmacy | Collins CD, et al/ 2020 | USA | A retrospective descriptive cohort on interventions done by pharmacists for patients with COVID-19 | 15 days | Patients with COVID-19 | Prospective audit and feedback, Pharmacist-driven (duration optimization, adverse drug reaction monitoring, renal and hepatic dose adjustment, QT interval monitoring, drug-drug interactions), Antibiotic use monitoring, Guideline development, tool development, medication supply coordination, antimicrobial surveillance, | - Clinical pharmacists conducted prospective audits and feedback mechanisms. - Clinical pharmacists made an average of 1.5 AMS interventions, including dose adjustment, interactions, laboratory monitoring, and adverse drug reactions monitoring - Clinical pharmacists developed a COVID-19 bundle which included monitoring for COVID-19 treatments (e.g., monitoring of the corrected QT interval (QTc) for hydroxychloroquine, keeping track of the days of therapy (DOT) for antiviral/antibacterial to reduce unnecessary prolonged use, and monitoring hepatic/renal function for dose optimization). - Clinical pharmacists managed shortages in antimicrobials, and were performing daily antimicrobial surveillance - Clinical pharmacists, students, and pharmacy residents participated in guideline development. - Clinical pharmacists recommended against the use of hydroxychloroquine and azithromycin.   It was noted that 15.4% of pharmacist interventions were for antimicrobial and COVID-19 treatment adjustment. Those included: treatment simplification, dosing adjustment, and timing adjustment. Additionally, antimicrobial surveillance, prospective audit and feedback were employed. | Patients received antimicrobial agents for relatively short durations (an average of 2.1 days for vancomycin, and 3  days for antipseudomonal antimicrobials) |
| 8 | Prescribing practices of lopinavir/ritonavir, hydroxychloroquine and azithromycin during the COVID-19 epidemic crisis and pharmaceutical interventions in a French teaching hospital | Gourieux B, et al/ 2021 | France | A descriptive study of the prescribing patterns of lopinavir/ritonavir, hydroxychloroquine, and azithromycin in COVID-19, and of the impact of the pharmaceutical interventions on it. | 1 month | Adults with COVID-19 who received at least one dose of lopinavir/ritonavir, hydroxychloroquine, or azithromycin | Guideline development, Pharmacist-driven interventions (dosing optimization, duration optimization, disease-drug interactions, drug-drug interactions, QT interval monitoring), | - A group of expert clinical pharmacist, hygienists, virologists, and infectiologists developed a treatment guideline for COVID-19 that was updated regularly. It included information on treatment regimens, interactions and monitoring - Pharmacists documented 59 interventions. Those included: drug-drug interactions, drug-disease interactions, dosing errors, and duration errors. - The most common errors were inappropriate duration (54.2%), Torsadogenic cardio-toxic interactions (23.7%), and inappropriate dosing (10.2%). | -81.4% of interventions were accepted by physicians.  -Response to interventions about teratogenic drug reactions (n=14) resulted in: implementing electrocardiographic (ECG) monitoring (n=7), discontinuation of drug (n=6), or in switching the drug (n=1). |
| 9 | On-ward participation of clinical pharmacists in a Chinese intensive care unit for patients with COVID-19: A retrospective, observational study | Wang R, et al/ 2021 | China | A description of the experience on medication optimization in a Chinese hospital ward for patients critically ill with COVID-19 | 1.5 months | Patients with COVID-19 admitted to an ICU ward | Pharmacist-driven (dose optimization, duration optimization, adverse reaction monitoring, therapeutic drug monitoring) | - Clinical pharmacist made 111 interventions. Most were related to antimicrobial agents. Of which 57.5% were related to antibacterial agents, and 47.7% to antiviral agents. - The most common interventions related to antibacterial and antifungal agents were:  1) Untreated indication. 2) Inappropriate drugs. 3) Under dosing. - Most common interventions related to antiviral agents were  1) Cardiovascular system. 2) Gastrointestinal tract.  3) Myelosuppression.  4) Liver function. | -95.5% of interventions were accepted by physicians.  -Most of the interventions related to antibacterial and antifungal agents led to drug discontinuation, followed by drug switching, dose adjustment, and then adverse drug reactions monitoring.  -For Antiviral drugs, the majority of the interventions led to drug discontinuation, followed by adverse drug reaction monitoring, and then drug switching. |
| 10 | Trends in antibiotic use before and during the coronavirus disease 2019 (COVID-19) pandemic across an integrated health system with different antimicrobial stewardship program models trends in antibiotic use by ASP model | Peterson JM, et al/ 2022 | USA | An analysis of antimicrobial stewardship program models (A, B, and C) and on the trends of antimicrobial use before and during COVID-19 The data were categorized into two periods (before COVID-19 and during COVID-19) | 2012-2022 | 2 years (before and during the pandemic) | Culture review, pharmacist-driven | In model C, which was pharmacist-led, a clinical pharmacist and an ID pharmacist performed daily reviews of positives cultures and made interventions which were communicated to the team through message or phone calls. | -The average monthly percent change (AMPC) of piperacillin-tazobactam (+0.6, p-value<0.1), and ceftriaxone (+0.9, p-value<0.1) increased, whereas the AMPC of vancomycin decreased (-0.3, p-value, 0.05) *However, those trends were also witnessed before the pandemic. |
| 11 | Interprofessional Collaboration between ICU Physicians, Staff Nurses, and Hospital Pharmacists Optimizes Antimicrobial Treatment and Improves Quality of Care and Economic Outcome | Schmid S, et al/ 2022 | Germany | Clinical performance data and consumption figures for antibiotics were analyzed over a 10-year period from  2012 to 2021. Period between 2012-2014 represent the control period before the implementation of ABS at MICU. Then the period between 2015-2021 represent data after the implementation of ABS at the MICU. |  | Medical intensive care unit at German University hospital | Pharmacist-driven (dose optimization, drug interaction, side effect monitoring) | Interprofessional team consisted of ICU physicians, pharmacists, and nursing who were responsible for the use of broad-spectrum antibiotic in term of (i) indication and selection of therapy, (ii) optimization of dosing, (iii) drug interactions, (iv) side effects, and (v) pharmacokinetic, pharmacodynamic, and pharmacoeconomic issues. | -The consumption of antibiotics increased during the pandemic in 2020 to 155.4 RDD/100 PD compared to the consumption in the years between 2015-2019 which was 132.5 RDD/100 PD. --Increase in total expenditure on antibiotics to 76,764 EUR due to the increase in numbers of severely ill COVID-19 patients. *However, in 2021 the consumption declined to 147.8 RDD/100 PD and the expenditure decreased to 75,292 EUR. |
